# Integrated Impact Assessment of Active Travel: Expanding the Scope of the Health Economic Assessment Tool (HEAT) for Walking and Cycling

**DOI:** 10.3390/ijerph17207361

**Published:** 2020-10-09

**Authors:** Thomas Götschi, Sonja Kahlmeier, Alberto Castro, Christian Brand, Nick Cavill, Paul Kelly, Christoph Lieb, David Rojas-Rueda, James Woodcock, Francesca Racioppi

**Affiliations:** 1 School of Planning, Public Policy and Management, University of Oregon, Eugene, OR 97403, USA; 2 Department of Health, Swiss Distance University of Applied Science (FFHS), CH-8105 Regensdorf, Switzerland; sonja.kahlmeier@ffhs.ch; 3 Epidemiology, Biostatistics and Prevention Institute, University of Zurich, CH-8001 Zurich, Switzerland; alberto.castrofernandez@swisstph.ch; 4 Transport Studies Unit, University of Oxford, Oxford OX1 3QY, UK; christian.brand@ouce.ox.ac.uk; 5 Centre for Exercise, Nutrition & Health Sciences, School for Policy Studies, University of Bristol, Bristol BS8 1TZ, UK; nick@cavill.net; 6 Physical Activity for Health Research Centre, Institute of Sport, Physical Education and Health Sciences, University of Edinburgh, Holyrood Road, Edinburgh EH8 8AQ, UK; p.kelly@ed.ac.uk; 7 Ecoplan AG, CH-3011 Bern, Switzerland; lieb@ecoplan.ch; 8 Environmental and Radiological Health Sciences, Colorado State University, Fort Collins, CO 80523, USA; david.rojas@colostate.edu; 9 Centre for Diet and Activity Research (CEDAR), MRC Epidemiology Unit, University of Cambridge School of Clinical Medicine, Cambridge CB2 0QQ, UK; jw745@medschl.cam.ac.uk; 10 WHO European Centre for Environment and Health, Platz der Vereinten Nationen 1, 53113 Bonn, Germany; racioppif@who.int

**Keywords:** active transportation, health impact assessment, physical activity, air pollution, traffic safety, carbon emissions, monetization, online tool

## Abstract

The World Health Organization’s Health Economic Assessment Tool (HEAT) for walking and cycling is a user-friendly web-based tool to assess the health impacts of active travel. HEAT, developed over 10 years ago, has been used by researchers, planners and policymakers alike in appraisals of walking and cycling policies at both national and more local scales. HEAT has undergone regular upgrades adopting the latest scientific evidence. This article presents the most recent upgrades of the tool. The health impacts of walking and/or cycling in a specified population are quantified in terms of premature deaths avoided (or caused). In addition to the calculation of benefits derived from physical activity, HEAT was recently expanded to include assessments of the burden associated with air pollution exposure and crash risks while walking or cycling. Further, the impacts on carbon emissions from mode shifts to active travel modes can now be assessed. The monetization of impacts using Value of Statistical Life and Social Costs of Carbon now uses country-specific values. As active travel inherently results in often substantial health benefits as well as not always negligible risks, assessments of active travel behavior or policies are incomplete without considering health implications. The recent developments of HEAT make it easier than ever to obtain ballpark estimates of health impacts and carbon emissions related to walking and cycling.

## 1. Introduction

Active travel modes, such as walking and cycling, are gaining broader consideration not only for their potential to alleviate problems of concern for transport and urban planners, but also as part of strategies to promote physical activity and health, particularly with regards to the prevention of non-communicable diseases, as well as mitigating climate change. As part of these developments, quantitative health impact assessments (HIAs) have become an important source of information for planners, policymakers and advocates alike. In this article we use the term health impact assessment in a narrow sense for quantitative assessments of health impacts. These may be part of broader health impact assessments using qualitative and/or quantitative methods for the purpose of policy evaluation, as for example described here: https://www.who.int/hia/en/. 

The World Health Organization’s (WHO) Regional Office for Europe has recognized the importance of integrating health considerations into transport appraisals, and in 2007 launched the first version of the Health Economic Assessment Tool (HEAT v1) for cycling. This was a spreadsheet-based calculator to assess health benefits in terms of premature deaths avoided due to physical activity from cycling [1,2]. Ever since, HEAT has been continuously developed by a collaborative team of researchers and practitioners under the umbrella of the WHO Regional Office for Europe (see Table 1). More recently, ongoing efforts have also involved the WHO Headquarters and the Pan-American Health Organization (PAHO). HEAT is now a staple in a small but growing family of HIA tools for active travel, along with, most notably, the Integrated Transport and Health Impacts Model (ITHIM, https://github.com/ITHIM), and a few others.

A main premise of the tool has been its usability, making it feasible to run a basic assessment in a short time, without any professional background in health, and based on minimal user inputs, while at the same time guaranteeing a robust scientific standard reflecting the latest research and the transparency of assumptions through an elaborate experts’ consensus process. It is worthy of note that, in many cases, HEAT is not only used by ‘traditional’ users of HIA, but also by planners or active travel advocates who are conducting their first economic assessment, in order to inform decision-making [3]. This makes it critical that HEAT is user-friendly and has a user interface that is as non-technical as possible. 

HEAT has been well received and applied across a large number of mostly European countries [3,4]. 

Early studies on the HIA of active travel indicated that the benefits from physical activity far outweigh the risks, such as exposures to air pollution, or injury risks from falls or crashes [5], and later work has confirmed this for the most part [6,7,8], albeit with some notable exceptions where crash risks outweighed benefits [9,10]. Based on such findings, and with the main objective of complementing existing transport appraisal methods, which usually did not include benefits derived from travel-related physical activity, HEAT up until version 3 was limited to the assessment of benefits of physical activity only [1,11]. While justifiable from a purely quantitative perspective, many users lamented that this left HEAT open to the criticism of being incomplete or selectively focused on benefits only. From a public health perspective, comparatively quantifying even small risks is worthwhile to support a more rational weighing of risks against benefits, and is potentially helpful in addressing some widely held misconceptions. Assessing risks related to active travel, such as those from air pollution exposure and traffic crashes, against the benefits of physical activity is challenging for both the public and decision makers, and how risks and benefits affect individuals’ travel decisions or policy decisions remains poorly understood. To fill part of this void, HEAT version 4 now integrates health impacts from physical activity, exposure to outdoor air pollution and crash risks (currently cycling only) for pedestrians and cyclists, as well as effects on vehicular carbon emissions from shifts away from motorized to active travel modes [12]. 

This increased versatility also led to increasing complexity. To preserve the tool’s user-friendliness, which is one of its most distinctive and appreciated features, a paradigm shift was applied to the tool’s architecture: rather than aiming to maintain a ‘one-size-fits-all’ approach, the tool flow and logic were revised fundamentally to be tailored to the user’s needs. A new module called “Your Assessment” now guides users to distinguish different assessment types, also referred to as use cases, accommodating a diverse set of the most common applications, using diverse data sources and formats. Another new module, called “Data Adjustment,” allows users to provide additional information, or to make educated guesses to further adjust their assessment, in case the data available to them are not ideal to conducting an assessment. 

The aim of this article is to present the updated methods and features of the latest HEAT version 4.2, as they apply to the integration of different exposures relevant to active travel into a single impact assessment tool, while balancing usability and scientific rigor. Scientific methods, technical approaches and aspired-for user experiences are discussed in the context of the broader progress made in HIAs of active travel, and some of the remaining challenges. 

Over the past three years, HEAT has undergone some substantial changes and additions, which are presented in this publication. HEAT is available at www.heatwalkingcycling.org.

## 2. Materials and Methods 

### 2.1. Overview and Rationale

**Figure 1:**
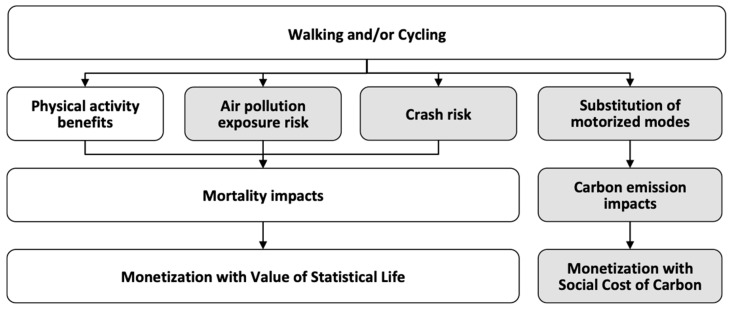
Impact pathways of the Health Economic Assessment Tool for walking and cycling (HEAT). Newly added pathways are highlighted in grey.

HEAT estimates the impacts on mortality and carbon emissions resulting from specified amounts of walking and/or cycling in a given population over a specified time period (see Figure 1).

#### 2.1.1. Basic HEAT Approach: Comparative Risk Assessment

**Figure 2:**
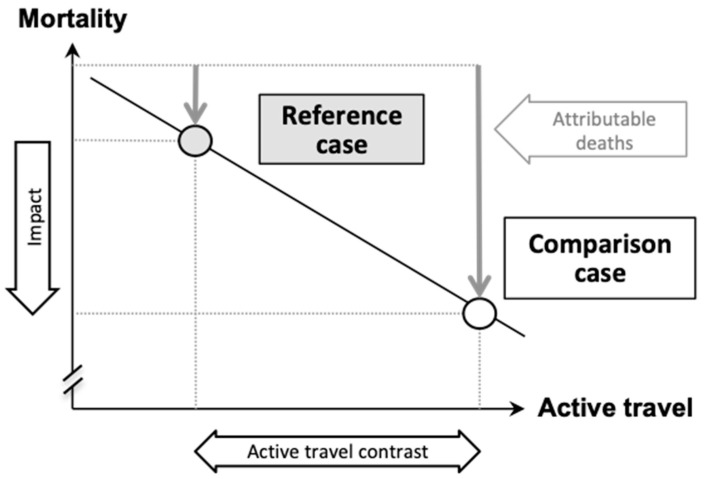
Schematic illustration of comparative risk assessment approach. Illustration depicts an example where increase in active travel from *reference case* to *comparison case* reduces mortality, i.e., is beneficial. Thus, attributable deaths in this case are “prevented deaths”. The top dotted line depicts the mortality level for a case without any active travel. In the *reference case* with lower active travel, fewer deaths are prevented, while in the *comparison case* with higher active travel more deaths are prevented due to active travel (Note: HEAT applies linear dose–response functions for physical activity, air pollution and crash risks).

HEAT applies a comparative risk assessment approach, based on aggregated, population-level data (i.e., population means). As such, the impacts of active travel are compared between two cases or scenarios, namely a *reference case* and a *comparison case* (also referred to as “counterfactual”) (see Figure 2). (Note to the reader: to facilitate orientation in the online tool, key terms used verbatim in the tool are italicized in this section).

There are two main assessment types. A *single case assessment,* or so-called steady state or status quo assessment, where the *comparison case* is set to zero, i.e., no walking or cycling, and a *two-case assessment*, where both the *reference case* and the *comparison case* are specified.

For each case, the levels of active travel are specified, and premature deaths (and/or carbon emissions) attributable to active travel (caused by the risks or avoided by the health benefits) are calculated. The impacts are the differences between the attributable deaths (and/or carbon emissions) in the *comparison case* minus the attributable deaths (and/or carbon emissions) in the *reference case* (HEAT ignores the impacts of other factors that may affect mortality over time, i.e., baseline mortality rates are kept constant throughout an assessment).

Attributable deaths are calculated for the *health pathways* of physical activity, exposure to outdoor air pollution and crash risk, using pathway-specific dose–response relationships based on reviews of the scientific evidence. Carbon emissions are calculated by assessing the effects on emissions due to shifts between motorized and active travel modes.

#### 2.1.2. Temporality in HEAT

The difference in active travel between *reference* and *comparison case* is first and foremost treated as time-independent. As such, the *comparison year* can be the same as, after or before the *reference year*, without any (major) effect on the impact calculation (the tool will use year-specific background data for some parameters). The user is, however, asked to specify the *assessment time* (i.e., number of years), such as over how many years the impacts should be calculated. *Assessment time* functions as a basic multiplier of annual impacts. However, there are several occasions where proper aspects of temporality are applied to the calculation, as follows:

(1) “*Uptake time*” specifies how long it takes to reach the maximum contrast in active travel levels (i.e., from reference to comparison levels). This considers the notion that once a new intervention/policy is established, it will take some time before it unfolds its full potential in increasing active travel. The tool sets up a year-sequence and linearly interpolates corresponding active travel volumes. The default value of one year can be modified by the user;
(2) “*Build-up time*” is a pre-defined lag period over which travel volumes develop the full health impacts of physical activity and air pollution, and is set to 5 years, based on expert consensus [13]. The build-up of full-magnitude health effects is interpolated linearly over the build-up time;
(3) “*Change in crash risk*” allows the user to specify a lower or higher crash risk for the *comparison case*, which the tool will interpolate linearly over the assessment period;
(4) “*Economic discounting*”, finally, adjusts the monetized annual impacts to their actual values in a user-defined year (by default the current year), considering the economic notion that benefits in the more distant future are of lower value than benefits that occur in the present or less distant future.

**Figure 3:**
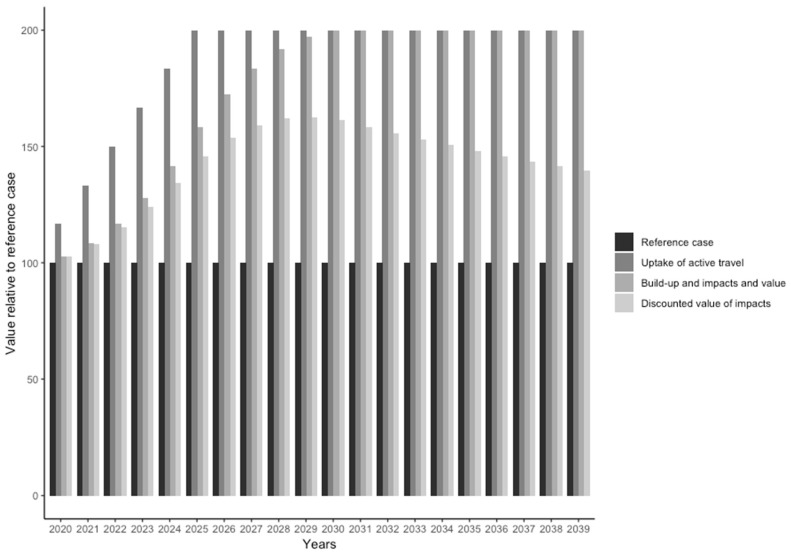
Schematic illustration of the temporal sequence in a HEAT assessment for a doubling of active travel over 20 years, relative to a *reference case* set to 100 (arbitrary unit). Over five years of *uptake time*, the *comparison case* will reach 200. The *build-up* of health impacts, and therefore the value of impacts, reaches the maximum after 5-year period (i.e., in 2030), and stays constant thereafter. The last category displays the economic values of impacts discounted to year 2020 values.

*Uptake* and *buildup time* are not applied to *single-case assessments*, which are treated as steady-state situations. Figure 3 illustrates the temporal aspects of a HEAT assessment.

### 2.2. Tool Features

**Figure 4:**
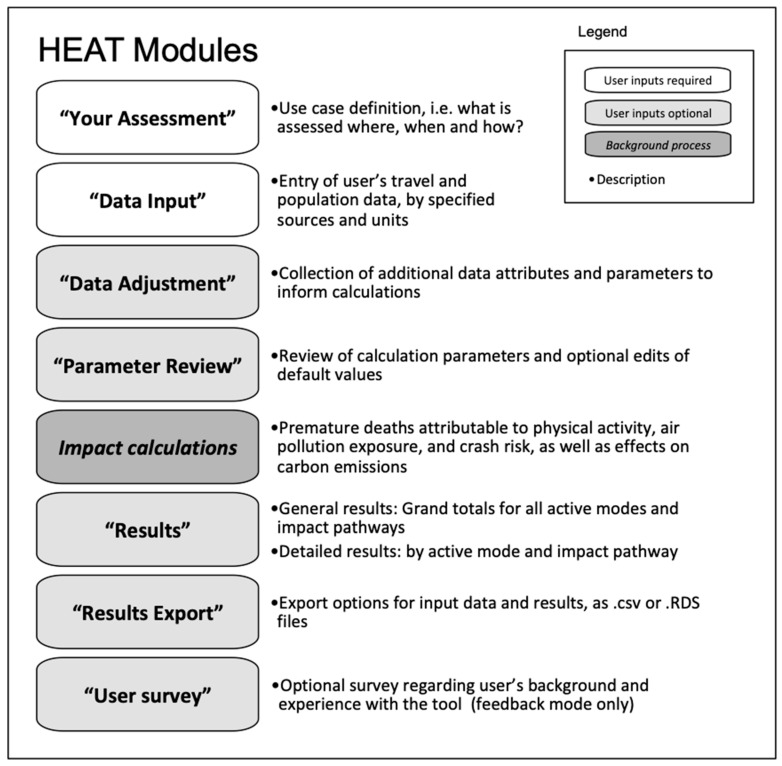
Flow of the Health Economic Assessment Tool for walking and cycling.

The structure of HEAT is illustrated in Figure 4. The main modules are described below.

#### 2.2.1. Use Case Definitions

HEAT is based on a flexible code structure with the ability to allow users to experience the simplest possible assessment process, tailored to their specific case study. To achieve this, the “use case” concept commonly used in software development was adapted (https://searchsoftwarequality.techtarget.com/definition/use-case). In HEAT, a use case is defined by inputs provided by the user (based on their local case study), which trigger a set of generic tool features and define the data and methods to be used. The use case is a systematic collection of characteristics that affect the validity of an assessment. 

As part of the new module “Your Assessment”, the tool collects a set of use case criteria. Specifically, the user is asked to specify the following:

(1) *Active travel mode*(*s*) assessed, namely walking and/or cycling. For the future, additional modes, such as bike sharing and e-bikes, are under consideration;
(2) *Geographic scale* of the assessment. *Country* and *city* are specified, based on which the tool pulls data on mortality rates, air pollution levels, crash risks and carbon emissions from underlying datasets (to the extent available, see also Section 2.2.2). Currently, countries and cities within the 53 Member States of the WHO European Region are supported. An expansion to accommodate other, non-preset use cases is under way, including the option of global applications. The user can also specify to conduct a *sub-city assessment*, which refers to the evaluation of specific facilities, infrastructures, neighborhoods, or similar. This has some methodological implications. For example, count data can only be used for *sub-city assessments*, because the extrapolation from count data to area-wide (i.e., city- or country-wide) levels of active travel remains extremely challenging. Crash risk assessments, on the other hand, are only offered for cycling and for selected countries, because the availability of crash risk estimates is very limited. Similarly, carbon emission assessments are performed using country-specific travel activity, vehicle fleet and temperature data.
(3) *Comparison and time scale*, i.e., whether to conduct a *single-case* or a *two-case assessment*, and when and for how long the assessment takes place.
(4) *Impacts* that should be considered, namely for the physiological *pathways* of exposure to physical activity, air pollution, crash risks, and/or carbon emissions.
(5) *Motorized modes*, i.e., whether the user has travel activity data for motorized modes or wants to use default data (currently for carbon emissions only).

#### 2.2.2. Background Data

HEAT aims to spare the user the burden of gathering data as much as possible. Where available, *default values* are provided by the tool. These include, in particular:

- mortality rates by country and age ranges (i.e., 20–44, 45–64, 45–74, 20–64, 20–74);
- air pollution levels for countries and cities (ambient particulate matter of less than 2.5 or 10μm in median diameter, respectively (PM_2.5_ or PM_10_));
- road fatality rates per distance cycled in selected countries [14];
- carbon emission factors for various travel modes, by country, as projected until 2050 (derived from international databases such as IIASA’s GAINS model, and speed-emissions curves building on EEA’s COPERT V model);
- value of statistical life estimates [15], by country.

Various additional parameters are provided as further default values which can be overwritten by the user (e.g., average trip distance, mode shift shares from cycling to car/public transport/walking), while several *background parameters*, typically based on generalizable scientific evidence, are hard-coded into the tool (e.g., relative risk estimates for health effects). A detailed overview is available in the online user guide [16] and on the tool website [17].

#### 2.2.3. Data Input

As a minimum, HEAT only requires users to provide data on *active travel* level(s) and the size (and, if possible, nature) of the *population* exposed to these levels of active travel. Users are prompted by the tool to provide active travel and population data at a single time-point for steady state, or at two time-points, for two-case comparisons. First, users specify their *data source* among any of the following options:

- hypothetical scenario (hypothesized active travel levels);
- population survey (measured/observed active travel levels, e.g., from travel survey);
- intercept survey (location-based, recommended only for sub-city assessments);
- count data (location-based, recommended only for sub-city assessments);
- modeled data;
- app-based data (mobile phone/wearable device).

Based on the selected *data source* for active travel a selection of *input units* is offered, as follows:

- minutes (per person, per day);
- hours (per person, per day);
- kilometers (per person, per day);
- miles (per person, per day);
- trips (per person, per day);
- counts (continuous, short-term; per location, per day);
- steps (walking only; per person, per day);
- frequency categories (percent of population);
- mode share (percent of total travel by all modes).

The next input field requests the actual number for travel volume. Basic units (i.e., minutes, kilometers, etc.) require volumes per person per day, whereas other units require additional inputs. The mode share option, for example, requires the entry of a percentage figure (the mode’s share of total travel volume), a number for the total travel volume, as well as a unit (trips, minutes or kilometers). The count data option requires (average) counts per day, per location. Using predefined frequency categories, users can approximate a categorical survey question about mode-use frequencies.

An entry field for *population type* supports the user in selecting the correct *population size*. An assessment can be based on data for the *general population*, i.e., averaged across all members of the population assessed, including those who may not bike or walk, or it may be based on *pedestrians and/or bicyclists* only. The latter, for example, applies to count data.

For the population(s) assessed, three *age range* options are provided, as follows:

- young adults (20–44);
- average (20–64 for cycling, 20–74 for walking);
- older adults (45–64 for cycling, 45–74 for walking).

Based on the selected *age range*, the tool applies the corresponding baseline mortality risks. The user is then required to specify the *size of the population* assessed, corresponding to the specified *population type* and *age range*.

The tool then converts the data on active travel to minutes and kilometers per person per day, respectively, which serve as the standard units for all subsequent calculations. Conversions are based on default assumptions of average travel speeds, trip distances and the like, which can be overwritten by the user, as well as the population size [16].

#### 2.2.4. Data Adjustment

In the next step of a HEAT assessment, the standardized active travel volumes can be further adjusted, or split into sub-volumes, to accommodate different calculations, depending on the use case. For each option, *default values* are provided, based on evidence where available, or expert consensus [16]. Details are available on the tool website [17].

Users have several options for adjusting the overall volumes of active travel modes, as presented in their original data:

- they can exclude some active travel (e.g., if attributed to a general trend in active travel that is not related to the intervention of interest);
- they can account for some *build-up time* until the comparison levels of active travel are reached;
- they can correct count data for seasonal or geographic distortion;
- they can quantify to what extent active travel may substitute other forms of physical activity.

To calculate air pollution impacts, active travel volumes need to be crudely dichotomized by location with regards to prevailing pollution levels, as follows:

- *proportion in traffic* vs. *away from traffic* (informing pollution levels assigned to the *comparison case*);
- *proportion for transport purposes* vs. *leisure* (informing pollution levels in the *reference case*).

For carbon emission calculations, active travel volumes need to be attributed to other modes they replace, if any. Users are asked to specify the following:

- *proportion of new trips* (i.e., induced travel not replacing previous travel)
- *proportion of reassigned trips* (i.e., trips that previously took place on a different route by the same mode);
- *proportions shifted from other modes* (i.e., walking, cycling, and various motorized mode categories).

Thereafter, the two sets of adjusted travel volume data and population data, namely for the *reference case* and the *comparison case*, are combined with effect estimates, such as relative risks or incident rates, emission factors, and various other background data provided by the tool to calculate pathway-specific impacts.

#### 2.2.5. Impact Calculations

The latest version of HEAT allows for assessing the net benefits of active travel, including the benefits derived through physical activity minus the negative health impacts from increased exposure to air pollution while walking or cycling, and from the risk of fatal crashes (cycling only). At the same time, a module to estimate the amounts of carbon emissions avoided by walking and cycling was added as well to assess the wider health benefits of mitigating climate change [16].

##### Physical Activity

Physical activity, such as that derived from walking and cycling, is associated with numerous health benefits, including the reduction of risk for cardio-vascular disease, diabetes, certain cancers, as well as all-cause mortality, among other things [18].

HEAT uses relative risk estimates for the effects of walking and cycling on all-cause mortality (adjusted for other forms of physical activity), which are based on a meta-analysis of epidemiologic cohort studies. The estimates from the literature are scaled to the level of active travel in the *reference case* and the *comparison case*, respectively, using a linear dose–response function which is capped at a maximum level of risk reduction (i.e., 30% for walking, 45% for cycling) [19]. Using a population attributable risk formula [20], the proportions of all deaths occurring in the assessed population which can be attributed to active travel are estimated (see Equation (1)). The difference between attributable deaths in the *reference case* and those in the *comparison case* reflects the impact of active travel in terms of premature deaths avoided.

Equation (1): Formulas to calculate physical activity impacts of HEAT.

RR_HEAT_ = RR_Lit_ × (AT_HEAT_/AT_Lit_)

MR_pop_ = MR_e_ × P_e_ + MR_u_ × P_u_

RR_HEAT_ = MR_e_/MR_u_

P_u_ = 1 − P_e_

MR_u_ = MR_pop_/[1 − (P_e_ × (1 − RR_HEAT_))] ~= MR_pop_ (1)

MR_e_ = MR_pop_ × RR_HEAT_/[1 − (P_e_ × (1 − RR_HEAT_))] ~= MR_pop_ × RR_HEAT_

D_e_ = MR_e_ × POP_HEAT_

D_u_ = MR_u_ × POP_HEAT_

D_attributed_ = D_e_ − D_u_

where:

RR_HEAT_ = relative risk of death for active travel assessed in HEAT

RR_Lit_ = relative risk of death, from the literature [19]

AT_HEAT_ = exposure (e) in terms of active travel volume assessed in HEAT

AT_Lit_ = reference volume of active travel for the RR_Lit_

MR_pop_ = mortality rate in the general population (for a specified age range)

MR_e_ = mortality rate in the exposed population

P_e_ = Proportion of the general population that is exposed. By default, set to almost zero (i.e., 0.001)

MR_u_ = mortality rate in the unexposed population

P_u_ = proportion of the general population that is not exposed

D_e_ = deaths in the assessed population with exposure

POP_HEAT_ = the population assessed in HEAT

D_u_ = deaths in the assessed population without exposure

D_attributed_ = deaths attributed to the exposure assessed in HEAT.

##### Air Pollution

Air pollution is associated with adverse effects on the cardio-vascular and respiratory system, as well as all-cause mortality [21,22]. The air pollution module in HEAT is based on earlier health impact assessments of air pollution exposure in active travelers [23,24]. To quantify the effects on all-cause mortality due to excess exposure to PM_2.5_ from active travel in pedestrians or cyclists, HEAT uses a relative risk estimate for fine particulate matter (PM_2.5_) based on epidemiologic studies [25]. HEAT currently does not estimate the effects of mode shifts to active travel on air pollution concentrations or the related effects on the health of the general population.

When engaging in walking or cycling, subjects are potentially exposed to higher intakes of air pollution mainly due to increased ventilation rates. HEAT assumes mode-specific constants for increased ventilation rates (1.37 m^3^/hour for walking and 2.55 m^3^/hour for cycling [26]). The air pollution concentration during walking or cycling is estimated based on simple assumptions applied to two user inputs: *proportion in traffic* determines which amount of active travel is taking place in an area of elevated air pollution concentration, for which a city’s background level is multiplied by a constant factor of 1.6 or 2, for walking or cycling, respectively [27]. For the *proportion away from traffic*, background levels are assumed. In addition, the user provides the *proportion of active travel which is for transport purposes* (vs. leisure). Active travel for transport in the *comparison case* is assumed to replace other means of transport, and therefore exposure to concentrations in traffic are applied to the *reference case*. Active travel for leisure (in the *comparison case*) is assumed to replace exposure to background levels (in the *reference case*). Based on this information, HEAT estimates an equivalent long-term average excess intake of air pollution which is used to scale the relative risk estimate from the literature to the local level. Using the same attributable risk formula as for physical activity, premature deaths attributable to air pollution exposure during active travel are calculated (see Equation (2)).

Since the studies on walking and cycling that are used to estimate the effects of physical activity on all-cause mortality did not exclude the concurrent effects on mortality of exposure to air pollution while being physically active, adjusted relative risk estimates for physical activity excluding the effects of air pollution are used when both pathways are assessed simultaneously. However, the differences are negligible [16].

##### Crash Risk

The risk of being involved in a crash while cycling or walking is an important adverse effect of active travel. Not only can it lead to great harm for an individual, but it may also deter people from walking or biking, and as such foregoing the often considerable health benefits associated with active travel [28]. However, estimating crash risks is often hampered by a lack of data. Exposure-adjusted crash risks, such as rates of expected crashes per kilometer, or similar, are rarely readily available. HEAT provides national rates for cyclist fatalities per kilometers traveled, for a number of countries [14]. The same rates are used for cities within these countries, with the option for the user to overwrite these with locally available data. The crash rates are multiplied with the provided volumes of active travel to calculate fatalities attributable to active travel. Users also have the option to specify the change in crash risk from *reference case* to *comparison case*, to reflect improvements in safety that may go along with, or may have led to, an increase in active travel.

Crash rates for pedestrians are currently not provided due to limited data availability. HEAT currently also does not support assessments of crash risks at the sub-city level, since reliable estimates of crash risks are very difficult to obtain at such scale [29]. Further, HEAT does not consider who is at fault in a crash, or any other crash attributes for that matter.

##### Carbon Emissions

Active travel is a low-emission form of transport, which has triggered great interest in the quantification of its potential to contribute to carbon reduction goals [30].

To estimate reductions in carbon emissions, HEAT requires users to provide additional information so as to qualify the assessed volumes of active travel with regards to the substitution of motorized modes. In the most comprehensive assessment, users provide travel volume data for all modes, including motorized, for both *comparison cases*. However, in the absence of such data, users can choose to provide simplified *proportions of new trips*, *proportions of reassigned trips* (for sub-city level use cases), and *proportions of active travel shifted from specific motorized modes*. For *single-case assessments*, the latter are sufficient. Further, users can specify local *traffic conditions* to provide a more accurate assessment of emissions savings. For all these parameters, *default values* are offered.

Carbon emissions are then calculated related to in-use (operational), energy supply and vehicle life-cycle emissions, applying extensive country and year-specific background parameters for fleet composition, fuel mix, trip lengths, ambient temperature and other relevant factors. Detailed descriptions and formula are available in the HEAT user guide [16].

#### 2.2.6. Impacts Aggregation, Monetization and Results Presentation

Temporal adjustments (as mentioned in Section 2.1.2) result in a sequence of impact estimates—one for each year throughout the assessment period.

Health impacts in terms of premature deaths prevented (or caused) are then monetized using the value of statistical life (VSL), a standard metric commonly used by transport planners [15]. By default, HEAT applies a base value of USD 3.0 million in 2005 from a review of 28 studies, including 261 VSL estimates. Country-specific default values are then derived, taking into account local GDP and income elasticity [16].

Equation (2): Formulas to derive country-specific values of statistical life from base value from Organization of Economic Collaboration and Development (OECD) [15].

VSL_country,2015(local currency)_ = VSL_OECD,2005,USD_ × (Y_country,2005_/Y_OECD,2005_)^IE^ × PPP_2005_ × (1 + %ΔP_2005–2015_) × (1 + %ΔY_2005–2015_)^IE^ (2)

where:

VSL_OECD,2005,USD_ = base value for OECD average of USD 3.013 million [15]

Y_country,2005_ = real gross domestic product (GDP) per capita at purchasing power parity in 2005 of the respective country (from http://search.worldbank.org/data)

Y_OECD,2005_ = OECD average real GDP per capita of USD 30,801 at purchasing power parity in 2005 (from http://search.worldbank.org/data)

IE = income elasticity of VSL of 0.8, according to OECD [15]

PPP_2005_ = exchange rate adjusted for purchasing power parity in 2005 (local currency per USD) (from http://search.worldbank.org/data)

1 + %ΔP_2005–2015_ = inflation adjustment with consumer price index of the respective country between 2005 and 2015

1 + %ΔY_2005–2015_ = income adjustment with growth in real GDP per capita in the respective country between 2005 and 2015

Climate change impacts of carbon emissions are monetized using social cost of carbon (SCC), a monetized value of the worldwide damage caused by the incremental impact of an additional ton of carbon dioxide equivalent (CO2e) emitted at a specific point in time [16]. Changeable default values for the SCC are provided by country and year, based on international evidence, regional averages or country-specific values (if available). SCC values for countries or contexts not covered in the existing evidence or policy guidance are allocated the European Commission recommended values (USD_2015_ 44 in 2015 rising to USD_2015_ 66 by 2030).

Monetized values in future years are then discounted (and values in the past are inflated) to reflect present values for a user-specified discount year.

The annual impacts are then summed up and presented separately by *active mode* and *pathway*, *active mode or pathway*, and *all active modes and pathways combined*. The results are presented as the annual average and total impacts over the assessment period. If users provide investment costs, benefit–cost ratios are calculated based on the total value of impacts.

### 2.3. Technical Implementation

In its latest version, HEAT was migrated to R Statistical Software, using R’s Shiny package, which “makes it easy to build interactive web apps straight from R” [31].

HEAT is set up through several distinct repositories in GitHub (www.github.com), including one for the R-code, one for the webapp (i.e., the user interface) and one for the website.

The R-code is structured like a self-standing R package. The webapp is set up to be built or modified through a spreadsheet template, allowing non-programmers to define details in the content and flow of the tool. Code developed by a professional programmer translates the spreadsheet into the actual tool. Shiny allows for maintaining a familiar, web-based user experience. The user interface only presents required input fields in a manner conditional upon previous entries (i.e., use case criteria), and rejects invalid entries. The new technical features also include the ability to export input data and results, and to provide the development team with direct in-tool feedback.

The website is also built using Shiny, and, similarly to the webapp, uses inputs from text files and spreadsheets to produce the contents.

Aside from hosting the code, GitHub is used to manage tool development and as an archive for institutional knowledge.

### 2.4. User Testing and Refinement

Throughout its development, HEAT has been based closely on the needs of existing and potential users—notably transport planners and policymakers. For this reason, the new version of HEAT was extensively tested with users before launch. Testers were recruited through the HEAT website and through conference presentations, and were invited to comment on new modules. A feedback page was built into the test site to enable live comments. This feature remains so that HEAT continues to be user-driven. According to Google Analytics, over the past 12 months alone (1 June 2019–31 May 2020) HEAT attracted over 6000 users, over 900 of which engaged with the website or tool for 10 min or more. Despite originally being developed for the European region, it has been used worldwide. A global version of the tool is currently being developed and will be available through the HEAT website in 2021.

The HEAT methodology, and in particular a broader review of the literature, HIA methods and the tool development process are described in the HEAT Methodology and User Guide [16]. HEAT is not an open source project, but those interested in collaborating are invited to contact the HEAT team.

## 3. Discussion

HEAT has been endorsed by government agencies in several countries [32,33]. In others, it has paved the way for the inclusion of active travel in official transport appraisals [10,34,35]. As arguably its most distinct feature, compared to other HIA tools or models available [7,23,36], HEAT aims to make HIAs as simple and feasible as possible, even for users with very little expertise in the area of travel behavior (data) and related health effects. With the most recent updates, HEAT has made a big step towards more comprehensive, integrated HIAs of active travel, addressing some of the most commonly expressed needs of its audience of policy makers, advocates, researchers, and urban and transport planners and practitioners alike.

By carefully applying a transparent approach to tool development, applied methods and assumptions, and a user-friendly web interface, HEAT provides an important tool to support the necessary political discourse around the value of investments in active travel. HEAT is well suited to initiate a conversation, raise awareness of the pros and cons of certain scenarios, or support an early case for or against a planned project. It aims to produce ‘ballpark estimates’ within the correct order of magnitude, but ambitions for accuracy much beyond that have been limited, in particular if it would come at the price of undermining user-friendliness and require more complex data inputs. As such, the key merits and applications of HEAT are as follows:

- Assessments of status quo, comparisons or changes of walking and/or cycling levels;
- Assessments at national, city or local project level;
- Optional consideration of motorized modes;
- Assessment of health impacts in terms of mortality due to physical activity, exposure to air pollution and crash risk;
- Assessment of impacts on carbon emissions;
- Monetization of impacts.

Recognizing and implementing the latest scientific developments has driven HEAT development over the past 10 years. Doing so within the constraints of a simple and generalizable tool thereby poses some particular challenges. For example, information concerning local aspects affecting impacts either have to be available through public databases, or users have to be able to provide them with minimal burden.

Overcoming this challenge to the assessment of air pollution impacts on active travelers was made possible by combining WHO’s global database of background air pollution levels, with parameters related to contrasts between air pollution background levels and levels in various modes of transport, published in a recent review article [27]. As such, users only have to provide two additional parameters: the proportion of active travel taking place in traffic, and the proportion of active travel for transport purposes. However, the often-requested feature of assessing the effects of increased active travel on reductions in air pollution concentrations and the resulting effects on general population health remains elusive, because there is no generalizable formula to translate a reduction in motor vehicle emissions to air pollution concentrations.

The calculation of impacts on carbon require a more substantial set of background data, in particular context-specific emission factors for cars, buses and motorbikes, and the required user inputs can be more numerous than for the other modules. For example, users may wish to change the default values and provide information on motorized modes substituted by active travel, either through actual data for motorized modes, or by estimating proportions of active travel shifted from motorized modes. Still, an impact assessment that does not involve changing any of the default values of mode shift or traffic conditions can be expected to provide a reasonably accurate assessment in most cases.

Crash risk assessment, on the other hand remains a major challenge. Exposure-adjusted fatality rates, such as rates that put road fatalities for active travelers in relation to the traveled volumes related to active modes, are not routinely available from any major database. This reflects the lack of recognition of active transport as an equal mode of transport. Thus, for HEAT these had to be compiled in a laborious effort, often requiring specific data handling for individual countries [14]. As such, this module is currently only available for cycling in the European region. Efforts to expand this to walking, and other countries, are underway.

The recent major review and meta-analysis of value of statistical life by OECD [15] has provided a much more robust base for the VSL default value used in HEAT, and users are now provided with country-specific default values reflecting substantial contrasts in wealth. However, many users have pointed out that these figures are often higher than those routinely used by transport professionals in their country. In these cases, users are encouraged to use locally appropriate values.

Aside from the challenges of translating the scientific state of the art into a practical tool, the quality of the input data that users need to provide has a major influence on the quality and accuracy of HIAs conducted with HEAT. The newly revised user interface, and in particular the sections for defining the use case, entering the data and adjusting the data as needed, aims to guide users more thoroughly through data sources and formats, and how they relate to the assessed population. New distinctions in data units and possible adjustments, on the one hand, help improving accuracy, while on the other hand take on the didactical purpose of sensitizing users towards different data collection methods, their possible limitations, and their implications for HIA methodology.

As HEAT aims to keep the user-burden for scientifically robust HIAs as low as possible, some important limitations need to be acknowledged, as follows:

- HEAT still only assesses mortality. This is not for lack of evidence about the effects of active travel on morbidities, i.e., cardio-vascular diseases and others, but for the reason of keeping the tool simple. Users interested in assessing impacts on morbidities may consider more complex tools, such as the Integrated Transport and Health Impact Model (ITHIM) [37], or conduct their own custom analysis;
- HEAT only considers impacts on adult populations. HEAT excludes subjects younger than 20 years old from the analysis, because first of all, mortality in these age ranges is very small, and second, for that same reason, there are no studies available for the effects of physical activity on mortality in these age ranges. HEAT further excludes subjects older than 74 years (for walking) or 64 years (for cycling), because mortality risks increase dramatically in older age ranges. Including these would therefore be highly influential on the results and potentially inflate the benefits of active travel;
- While the aim is to enable users to use their data as much as possible in the format they collect or obtain them, the task of aggregating the active travel data to population averages for the two *comparison cases* is left to the user. As such, HEAT is indifferent to any non-linear patterns in active travel developments that may be captured by sophisticated data sources or models, such as, for example, agent-based models;
- HEAT only considers the effects for active travelers (albeit averaged across the general population). The effects of active travel on the general population, for example through lower air pollution or increased traffic safety, are not assessed;
- Crash risk assessments are only available for cycling in selected countries, and at the country or city level. Crash risk assessments at lower geographic scales, such as for specific infrastructures or road segments, are not considered feasible because the data collection or modeling required to obtain such crash risk estimates remains extremely challenging;
- HEAT currently only allows for a crude reflection of the age of the assessed population. Substantial deviations in the age of the assessed population compared to the specified age range may result in substantial inaccuracies.

Several future features for HEAT are currently being considered or are under development. These include, in particular, the accommodation of North American and other non-European use cases (with less preset default data available), a more refined handling of age and population data, and the expansion to new active modes, such as bike sharing and e-bikes, among others.

Equally as important as the features visible on the user interface is the technical set-up in the background. The shift to R-statistical software was motivated by the need to shift HEAT development in a more sustainable, collaborative and transparent direction. Bridging scientific background work with tool development and programming has been a continuous challenge in HEAT’s evolution. Initially published as a spreadsheet calculation, HEAT was moved to a web-based tool, mainly to facilitate access and dissemination, at the cost, however, of widening the gap between researchers developing the methods and programmers in charge of building the tool. An important aim of the recent update was to narrow this gap and move the tool building process closer to the research team, limiting the need for advanced programming skills as much as possible. Software tools like R, Shiny, Slack and GitHub have proven invaluable to creating a more sustainable development environment, but nonetheless further developing and maintaining HEAT remains a major effort.

Funding HEAT has proven to be extraordinarily challenging, quite possibly due to its translational nature, falling between the cracks of pure research and practical web-applications.

## 4. Conclusions

The recent developments in HEAT make it easier than ever for policymakers, researchers and practitioners to account for health benefits derived from physical activity and risks derived from exposure to air pollution and crashes, as well as impacts on carbon emissions, in their plans and projects affecting active travel.

HEAT is fully in line with current and forthcoming policy frameworks, including the Declaration of the Sixth Ministerial Conference on Environment and Health [38], the European Physical Activity Strategy for the WHO European Region (2016–2025) [39] and the Global Action Plan on Physical Activity and Health [40], as well as climate change policies globally, nationally and locally [41,42,43].

As active travel inherently results in often substantial health benefits as well as not always negligible risks, planning for active travel is incomplete without considering its health implications.

## Figures and Tables

**Table 1:** HEAT versions overview.

HEAT Version () = Early Versions Not Officially Numbered	(v1)	(v2)	(v3)	4.0	4.1	4.2
**Release Date**	2009	2011	2014	2017	2018	2019
**Description of new features**	na	- Walking - Web-based	- Updated relative risks for walking/cycling and all-cause mortality. - updated *Value of Statistical Life* (*VSL*) - introduced country-specific VSL values	- Combined walking and cycling. - New impact pathways: exposure to air pollution, crash risk, carbon emissions - Updated country-specific *Values of Statistical Life* (*VSL*) - revised tool workflow - new user interface	- Improved user experience (such as warning messages for invalid entries) - Data export feature. - User feedback feature	- Revised travel data input page - Revision of underlying R code
**Software platform**	Excel	Website/html	Website/html	R + Shiny	R + Shiny	R + Shiny
**UI features**	Spreadsheet	Webinterface	Webinterface	- Shiny webinterface - Conditional (“tailored”) workflow based on use case definition (“Your assessment”) - Module for adjustment of travel data inputs	- Validity blocks and warnings. - Data export to .csv and .Rds. - Feedback option for every page and integrated user survey.	- More intuitive travel data input.
**Geographic range**	WHO Europe	WHO Europe	WHO Europe	WHO Europe	WHO Europe	WHO Europe
**Modes included**	- Cycling	- Cycling - Walking	- Cycling - Walking	- Cycling - Walking	- Cycling - Walking	- Cycling - Walking
**Impact pathways**	- Physical activity	- Physical activity	- Physical activity	- Physicaly activity - Air pollution - Crashes - Carbon emissions	- Physicaly activity - Air pollution - Crashes - Carbon emissions	- Physicaly activity - Air pollution - Crashes - Carbon emissions
**Methodological changes**	- Linear dose-response function	- Log-linear dose response function	- Linear dose-response function with cap - Country-specific *Values of Statistical Life* (*VSL*) based on a methodology developed by the OECD (2012)			- Revised calculations of temporal sequences (uptake of active travel, buildup of health impacts, economical discounting)
**Documentation**	Rutter, H., 2007. Health economic assessment tool for cycling (HEAT for cycling) (Excel sheet). WHO Regional Office for Europe, Copenhagen. Rutter, H., Cavill, N., Dinsdale, H., Kahlmeier, S., Racioppi, F., Oja, P., 2008. Health Economic Assessment Tool for Cycling - User guide. World Health Organization Regional Office Europe, Rome. Cavill, N., Kahlmeier, S., Rutter, H., Racioppi, F., Oja, P., 2007. Economic assessment of transport infrastructure and policies: Methodological guidance on the economic appraisal of health effects related to walking and cycling. WHO Regional Office for Europe, Copenhagen. Cavill, N., Kahlmeier, S., Rutter, H., Racioppi, F., Oja, P., 2008a. Methodological guidance on the economic appraisal of health effects related to walking and cycling: summary. Economic assessment of transport infrastructure and policies. WHO Regional Office for Europe, Copenhagen. *WHO/Europe, 2007. Review of economic analyses of transport infrastructure and policies including health effects related to physical activity: Consensus workshop. Meeting report. 15–16 May 2007, Graz, Austria.*	Kahlmeier, S., Cavill, N., Dinsdale, H., Rutter, H., Gotschi, T., Foster, C., Kelly Clarke, D., P., Oja, P., Fordham, R., Stone, D., Racioppi, F., 2011. Health economic assessment tools (HEAT) for walking and for cycling. Methodology and user guide. WHO Regional Office for Europe, Copenhagen. *Rutter, H., Cavill, N., Racioppi, F., Dinsdale, H., Oja, P., Kahlmeier, S., 2013. Economic Impact of Reduced Mortality Due to Increased Cycling. Am J Prev Med 44, 89–92.* *WHO/Europe, 2010. Development of guidance and a practical tool for the economic assessment of health effects from walking. Meeting report. Consensus workshop. 1–2 July 2010, Oxford, UK.*	Kahlmeier, S., Kelly, P., Foster, C., Götschi, T., Cavill, N., Dinsdale, H., Woodcock, C., Schweizer, C., Rutter, H., LIeb, C., Oja, P., Racioppi, F., 2014. Health economic assessment tools (HEAT) for walking and for cycling: Methodology and user guide —updated reprint, 2014. *WHO/Europe, 2014. Development of the health economic assessment tools* (*HEAT*) *for walking and cycling. Meeting report of the consensus workshop in Bonn, Germany, 1–2 October 2013.* *WHO/Europe, 2014. Development of the Health economic assessment tools* (*HEAT*) *for walking and cycling. 4th Consensus meeting. Meeting report. Bonn, Germany, 11–12 December 2014.* *OECD, 2012. Mortality Risk Valuation in Environment, Health and Transport Policies.*	Kahlmeier, S., Götschi, T., Cavil, N., Castro Fernandez, A., Brand, C., Rojas Rueda, D., Woodcock, J., Kelly, P., Lieb, C., Oja, P., Foster, C., Rutter, H., Racioppi, F., 2017. Health economic assessment tool (HEAT) for walking and for cycling. Methods and user guide on physical activity, air pollution, injuries and carbon impact assessments (2017). World Health Organization, Regional Office for Europe. *WHO/Europe, 2017. Development of the health economic assessment tools* (*HEAT*) *for walking and cycling. 5th consensus meeting: meeting report. Copenhagen.*		
**URL**			http://old.heatwalkingcycling.org/			https://www.heatwalkingcycling.org
